# The Preservation of Umbilical Blood Supply in Combined Ventral Hernia Repair and Abdominoplasty: A Narrative Review

**DOI:** 10.1007/s00266-024-03999-y

**Published:** 2024-04-22

**Authors:** Marcel Chua, Ishith Seth, Vicky Tobin, Elan Kaplan, Warren Matthew Rozen

**Affiliations:** 1 Faculty of Medicine, The Alfred Centre, Central Clinical School, Monash University, 99 Commercial Rd, Melbourne, VIC 3004 Australia; 2 Department of Plastic and Reconstructive Surgery, Peninsula Health, 2 Hastings Road, Frankston, VIC 3199 Australia; 3 Department of General Surgery, Peninsula Private Hospital, 525 Mclelland Drive, Langwarrin, VIC 3910 Australia; 4 Monash Doctors Workforce, Monash Health, 246 Clayton Road, Clayton, VIC 3168 Australia

**Keywords:** Hernia repair, Abdominoplasty, Umbilicus blood supply, Ventral hernia, Abdominal anatomy, Abdominal wall repair

## Abstract

**Introduction:**

Combined ventral hernia repair and abdominoplasty treat risk factors such as high body mass index and weak abdominal musculature, providing excellent intraoperative exposure and improved patient outcomes. Unfortunately, a combination of traditional procedures is unfeasible as the umbilical blood supply would be compromised, leading to increased umbilical necrosis risk. This narrative review aimed to identify new techniques and solidify evidence in preserving umbilical blood supply and associated level of evidence.

**Methods:**

Two authors conducted a thorough literature search on PubMed, Scopus and Cochrane CENTRAL databases from January 1901 to July 2023, adhering to the methodologies of the preferred reporting items for systematic reviews and meta-analyses. Studies were reviewed for their surgical technique and quality of evidence. The primary outcomes of interest consisted of umbilical complications of this combined procedure.

**Results:**

Six techniques were identified that included laparoscopic, pre-rectus, unilateral, distal bilateral, proximal bilateral, and inferior midline approaches. All techniques demonstrated as viable options in preserving umbilical blood supply as reported complications were few, minor, and compounded by risk factors. However, all included techniques were limited to low-to-moderate-quality evidence.

**Conclusion:**

Despite the lack of high-quality evidence, all techniques remain viable options for combined ventral hernia repair and abdominoplasty. Large-scale high-quality RCTs are required to compare the effectiveness of various approaches with additional outcomes of hernia recurrence rates, intraoperative time, and patient- and surgeon-reported satisfaction.

**Level of Evidence IV:**

This journal requires that authors assign a level of evidence to each article. For a full description of these Evidence-Based Medicine ratings, please refer to the Table of Contents or the online Instructions to Authors www.springer.com/00266.

## Introduction

In the field of plastic surgery, the repair of ventral hernias associated with ventral myofascial defects due to high body mass index (BMI) or pregnancy conventionally involves abdominal wall reconstruction. Techniques have been previously described for such procedures, for example, the use of synthetic mesh repair, autologous options including the pedicled muscle or musculocutaneous flaps, or the closure of the defect with autologous dermal grafts [1, 2]. In the evolvement of these techniques, the repair of ventral hernias, a broad term for umbilical, epigastric, and incisional hernias, is being increasingly performed concomitantly with abdominoplasty [3–6]. By combining these procedures, risk factors for hernias such as high BMI can be addressed while maintaining the platform for abdominal wall reconstruction [6–10]. Additionally, combining these two procedures allows for excellent exposure to the hernia intraoperatively potentially improving patient outcomes [6, 11].

**Fig. 1:**
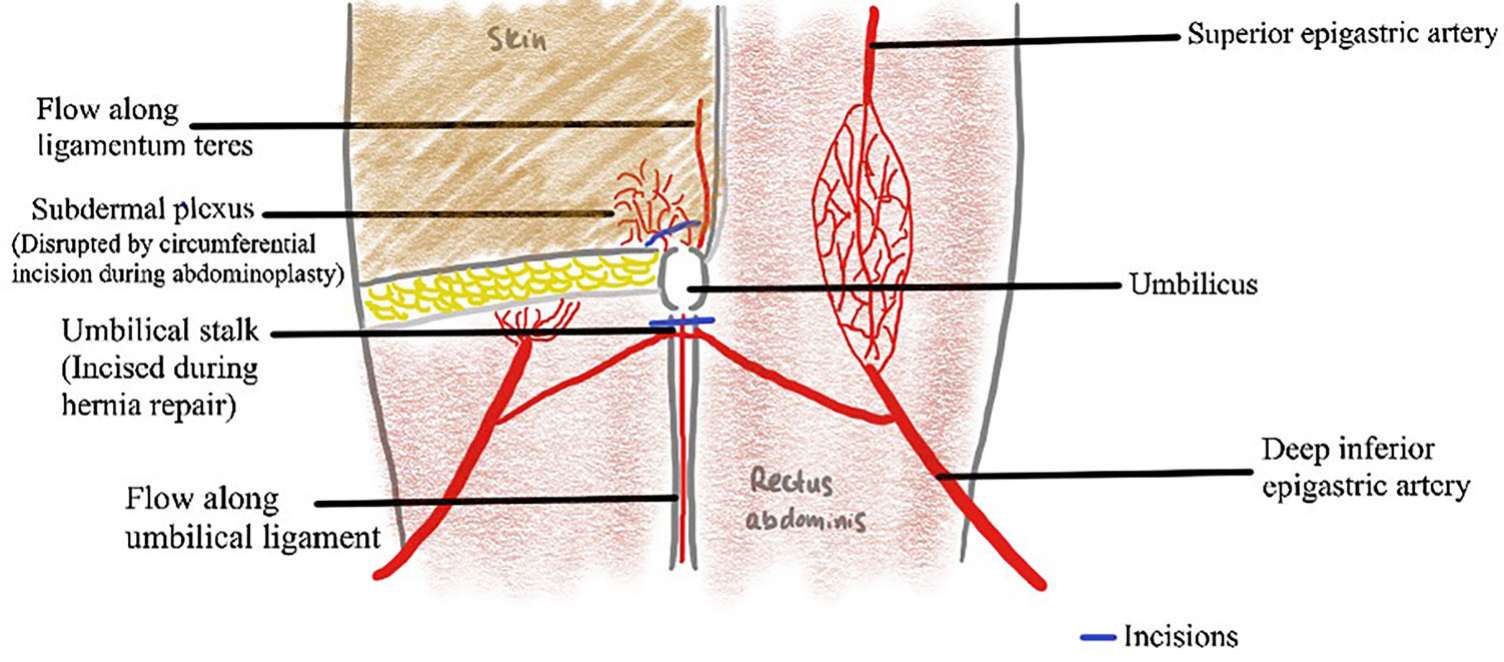
Illustration on the anatomy of the blood supply to the umbilicus

The combination of traditional open hernia repair and abdominoplasty has not been feasible due to the compromise of the umbilical blood supply. Upon review of umbilical anatomy (Fig. 1), its blood supply could be split into deep or superficial networks. The deep network consists of perforators of the deep inferior epigastric arteries, ligamentum teres hepaticum, and the median umbilical ligament, which all converge into the umbilical stalk [12–14]. The superficial network consists of the subdermal plexus [12–14]. Traditionally, open hernia repair transects the umbilical stalk while abdominoplasty interrupts the subdermal plexus by circumscribing the umbilicus to free it from the surrounding tissue [15–17]. This obliterates the blood supply to the umbilicus leading to an increased risk of umbilical necrosis [10, 13–16]. Therefore, developing a conventional technique that preserves umbilical blood supply would significantly improve patient outcomes.

This narrative review was aimed to identify all available surgical techniques in the literature and provide evidence of their effectiveness in preserving the umbilicus blood supply. This will assist in recommending safe and effective techniques and highlight the need for further evidence-based studies or innovation.

## Methods

PubMed, Scopus, and the Cochrane Library databases were searched for relevant articles by two authors published from January 1901 to July 2023, adhering to the methodologies of the preferred reporting items for systematic reviews and meta-analyses (PRISMA) [18]. The search terms included “ventral hernia”, “abdominoplasty” and “umbilicus” and were used in conjunction with a combination of Boolean operators AND and OR. The final search algorithm consisted of medical search headings (MeSH) terms including “hernia, ventral”, “abdominoplasty” and “plastic surgery procedures” and free-text term “umbilicus”. The last search was performed on 5 July 2023. Additionally, reference lists of all included articles and previous review articles were cross-referenced to include more articles.

All search results were exported into EndNote V21 and duplicates were removed. The titles and abstracts of remaining articles were screened and included in this review if they were: (a) English language, (b) describing a surgical technique in combined ventral hernia repair and abdominoplasty, and (c) studies of controlled trials, cohort studies, case series, or reports. Exclusion criteria were: (a) miniabdominoplasty, (b) preliminary reports, and (c) unpublished results from ongoing trials.

The articles selected for inclusion were first reviewed for their description of surgical techniques. Aspects of these procedures included the type of hernia repair, the plane of repair, the anatomical approach to the umbilicus, and the type of hernia closure. Additionally, the effectiveness and quality of evidence in preserving the umbilicus blood supply were assessed. The demographics of each study were included; study design, number of participants, sex, age, BMI, hernia type, presence of a comparison technique, and complications. Level of evidence was assigned according to the Oxford Center of Evidence-Based Medicine (OCEBM) 2011 [19].

## Results

### Literature Search

**Fig. 2:**
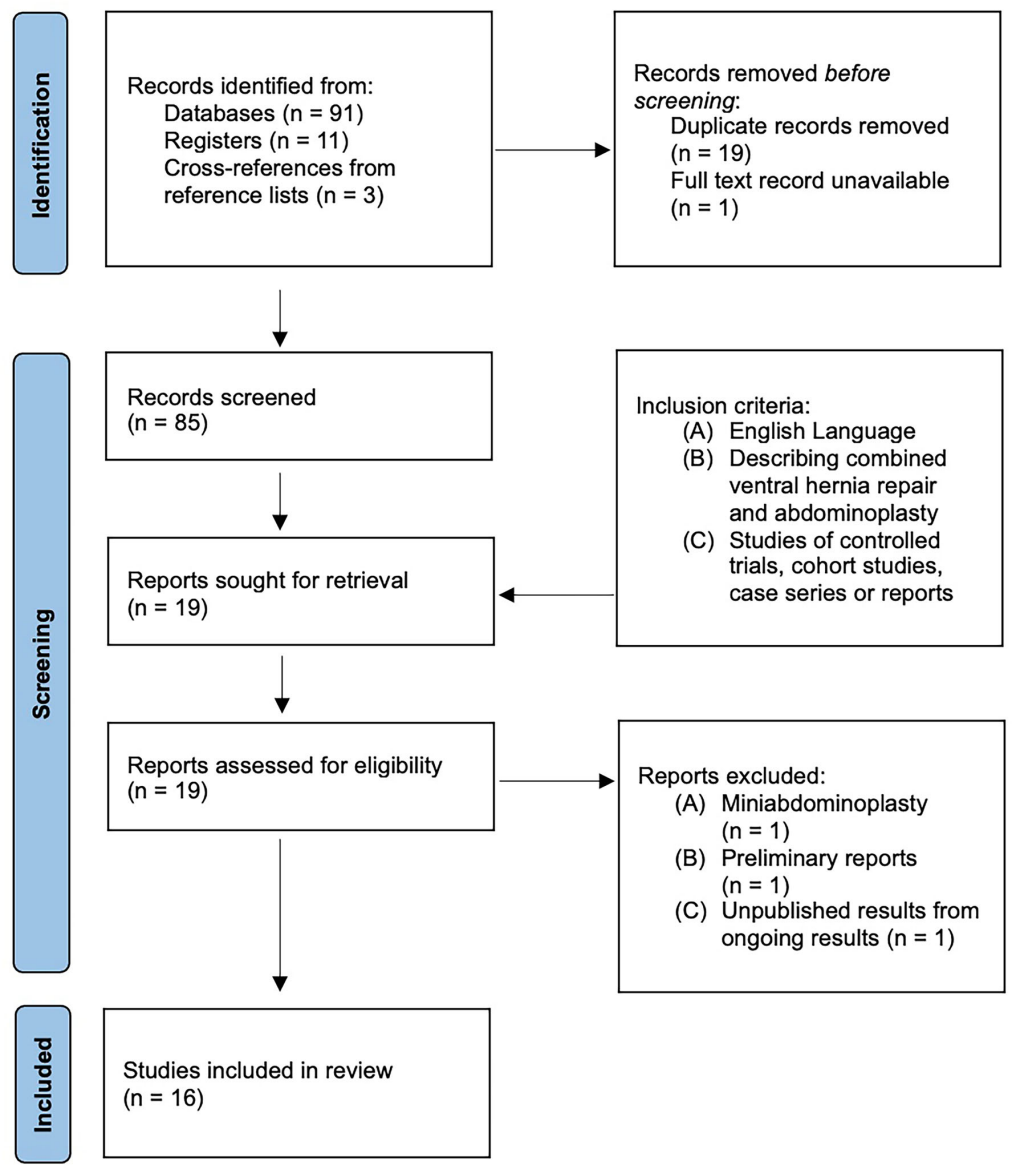
Preferred reporting items for systematic reviews and meta-analyses flow diagram of literature search and screening of studies eligible for review

The process of literature search is presented as a PRISMA flow diagram (Fig. 2). The initial search strategy yielded 105 articles. The final sixteen included articles comprised of two randomized controlled trials (RCT), two cohort studies, eight case series, and four case reports. Six unique techniques were identified, which included: (a) laparoscopic approach, (b) pre-rectus approach, (c) unilateral approach, (d) distal bilateral approach, (e) proximal bilateral approach, and (f) inferior midline approach.

### Surgical Techniques

**Fig. 3:**
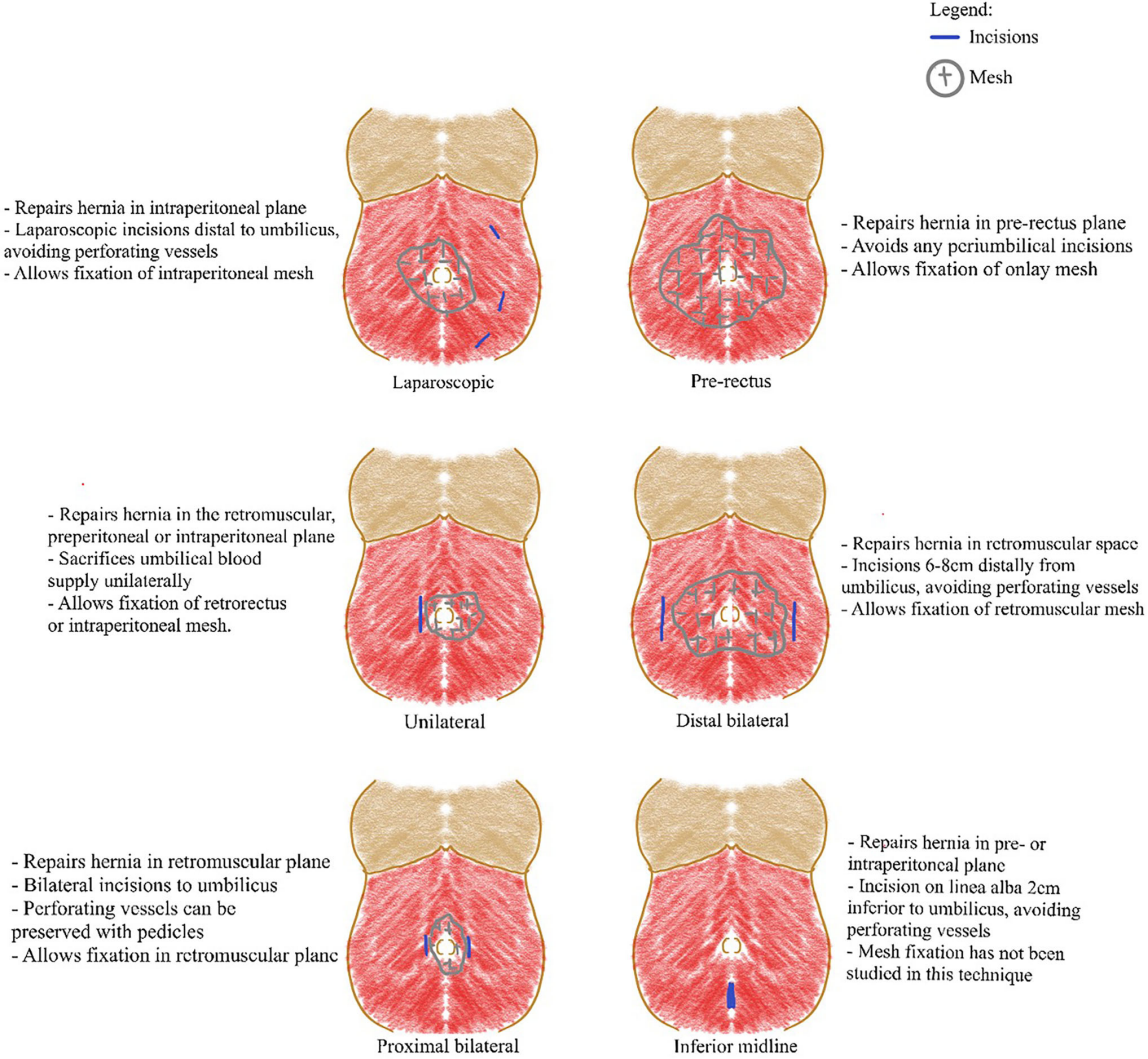
Illustration of techniques in combined ventral hernia repair and abdominoplasty

**Fig. 4:**
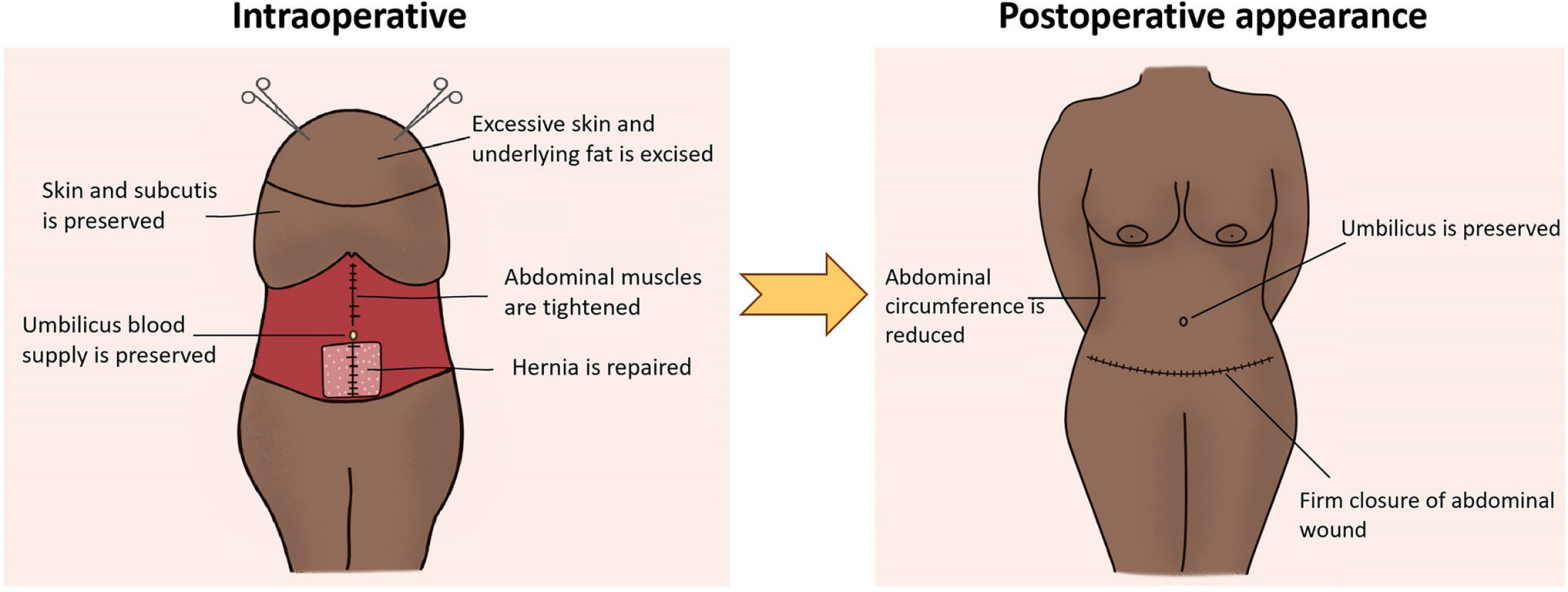
Schematic diagram of intraoperative and postoperative appearance of combined procedure

The breakdown of each technique’s process is detailed in Table 1 and illustrated in Fig. 3. Techniques differ in their anatomical approach to the umbilicus to access a desired plane for hernia repair as described below. Abdominoplasty following hernia repair was performed similarly across all techniques as illustrated in Fig. 4.

**Table 1:** Breakdown of surgical techniques in combined hernia repair and abdominoplasty [3, 6–11, 15–17, 20–25]

Study	Type of repair	Plane of repair	Anatomical approach to umbilicus	Type of hernia closure
*Laparoscopic approach*				
Halsey et al. (2021)	Laparoscopic	Intraperitoneal and anterectus	Laparoscopic (No periumbilical incisions)	Suture
Lari et al. (2019)	Laparoscopic	Intraperitoneal	Laparoscopic (No periumbilical incisions)	Intraperitoneal mesh
van Schalkwyk et al. (2018)	Laparoscopic	Intraperitoneal	Laparoscopic (No periumbilical incisions)	Intraperitoneal mesh
Phan et al. (2018)	Laparoscopic	Intraperitoneal	Laparoscopic (No periumbilical incisions)	Intraperitoneal mesh
Le Gall et al. (2017)	Laparoscopic	Intraperitoneal	Laparoscopic (No periumbilical incisions)	Intraperitoneal mesh
*Pre-rectus approach*				
Erfan et al. (2023)	Open	Pre-rectus	No periumbilical incisions	Suture
Zhitny et al. (2022)	Open	Pre-rectus	No periumbilical incisions	Suture
Sakr et al. (2019)	Open	Pre-rectus	No periumbilical incisions	Suture only or suture with onlay mesh
Eltantawy (2019)	Open	Pre-rectus	No periumbilical incisions	Suture and onlay mesh
Shermak et al. (2006)	Open	Pre-rectus	No periumbilical incisions	Suture
*Unilateral approach*				
Neinstein et al. (2015)	Open	Intraperitoneal	Unilateral	Intraperitoneal mesh
Mcknight et al. (2012)	Open	Preperitoneal	Unilateral	Retrorectus mesh
Iannelli et al. (2007)	Open	Retromuscular	Unilateral	Retrorectus mesh
*Distal bilateral approach*				
Hadidi et al. (2020)	Open	Retromuscular	Bilaterally 6–8 cm from midline	Retrorectus mesh
*Proximal bilateral approach*				
Moreno-Egea et al. (2016)	Open	Retrorectus	Bilateral (exact distance unspecified)	Retrorectus mesh
*Inferior midline approach*				
Bruner et al. (2009)	Open	Pre- or intraperitoneal	Inferior midline, 2 cm inferior to umbilicus	Suture

The laparoscopic approach was described by five studies, which accesses the intraperitoneal plane by inserting two to three laparoscopic ports, which are distal to the umbilicus and avoid disruption of the perforators coursing through the deep tissues [7, 16, 17, 20, 21]. The hernia could be closed by fixation of an intraperitoneal mesh in the intraperitoneal plane as described in four of five studies [7, 16, 17, 20]. The remaining study described hernia closure with two sutures: one in the intraperitoneal plane and the other in the pre-rectus plane [21].

The pre-rectus approach was described by five studies, in which the hernia was closed with sutures in the pre-rectus plane, thus avoiding any periumbilical incisions [6, 8, 9, 11, 22]. Two studies described the use of an onlay mesh to reinforce the abdominal wall integrity [8, 22].

The unilateral approach was reported by three articles, all describing incisions to the rectus aponeurosis on one side to access the retromuscular, preperitoneal or intraperitoneal plane, and subsequently the fixation of a retrorectus or intraperitoneal mesh. The unilateral incision sacrifices perforators on the side of the incision but preserves the contralateral side [15, 23, 24].

The distal bilateral approach, reported by a single study, was described to involve bilateral incisions 6–8 cm away from the midline to access the retromuscular space for mesh fixation. This avoids perforators that are located more proximally to the umbilicus [25]. The proximal bilateral approach, also reported by a single study, involved bilateral incisions proximal to the umbilicus to access the retrorectus space for mesh fixation; however, the incision distance was not reported [3]. Finally, the inferior midline approach, reported by a single study, described an incision 2 cm inferior to the umbilicus, through the linea alba to access the pre- or intraperitoneal plane [10].

### Effectiveness and Quality of Evidence

The effectiveness of each technique emphasized by the presence of umbilical complications is presented in conjunction with the assessment of evidence quality in Table 2. All techniques appear to be viable options for preserving umbilical blood supply and only five out of 16 studies reported cases of umbilical complications. Additionally, these complications were minor, occurred in low frequencies and had risk factors for poor healing (e.g., diabetes and obesity).

**Table 2:** Review of effectiveness and quality of evidence of surgical techniques [3, 6–11, 15–17, 20–25]

Study	Type of study	Population					Presence of comparison technique	Consideration of umbilical complications as an outcome	Reported umbilical complications	Level of evidence
		No. of subjects	Sex	Hernia type	Mean age	Mean BMI (kg/m^2^)				
*Laparoscopic approach*										
Halsey et al. (2021)	Case report	1	Female	Umbilical	32	–^a^	No	No	–^a^	V
Lari et al. (2019)	Case series	47	Male and female	Umbilical	35	26	No	Yes	None	IV
van Schalkwyk et al. (2018)	Case series	10	Female	Umbilical	37.2	23.8	No	Yes	None	IV
Phan et al. (2018)	Case report	1	–^a^	Incisional and inguinal	–^a^	–^a^	No	Yes	None	V
Le Gall et al. (2017)	Cohort study	15	Male and female	Umbilical and incisional	43.9	31.4	No	Yes	One reported superficial cutaneous umbilical necrosis.	IV
*Pre-rectus approach*										
Erfan et al. (2023)	Case series	15	Female	Paraumbilical, umbilical and epigastric	37.73	32.18	No	Yes	One reported umbilical infection and partial loss.	IV
Zhitny et al. (2022)	Case report	1	Female	Umbilical	38	27.4	No	No	–^a^	V
Sakr et al. (2019)	Cohort study	78	Female	All ventral types	41.37	32.2	No	No	–^a^	V
Eltantawy (2019)	Randomized controlled trial	50	Male and female	Paraumbilical, epigastric and incisional	40.1	36.6	No	Yes	Thirteen reported unpreserved umbilicus intraoperatively due to encroachment of hernia sac on umbilical stalk.	III
Shermak et al. (2006)	Case series	40	Male and female	Incisional	42	35.6	No	No	–^a^	V
*Unilateral approach*										
Neinstein et al. (2015)	Case series	11	Females	Umbilical	39.4	27.6	No	Yes	None	IV
Mcknight et al. (2012)	Case report	1	Female	Umbilical	–^a^	–^a^	No	Yes	None	V
Iannelli et al. (2007)	Case series	5	Female	Midline incisional	42.2	28.6	No	Yes	One reported partial necrosis (additional risk factors of diabetes and BMI of 35kg/m^2^). Another reported epidermal necrosis.	IV
*Distal bilateral approach*										
Hadidi et al. (2020)	Case series	67	Male and female	All ventral types	46	29.6	No	No	–^a^	V
*Proximal bilateral approach*										
Moreno-Egea et al. (2016)	Randomized controlled trial	111	Male and female	Incisional	66	33	No	No	At least two cases of umbilical excision and/or umbiliconeoplasty (numbers unspecified).	IV
*Inferior midline approach*										
Bruner et al. (2009)	Case series	17	–^a^	Umbilical	–^a^	–^a^	No	Yes	None	IV

^a^Not reported or not clearly defined

Overall, all case reports, case series, and cohort studies were of poor quality (level IV–V), while RCTs were of moderate quality (level III). There was no high-quality evidence in any studies (level I–II).

## Discussion

A previous review by Person et al. discussed the pros and cons of current methods for simultaneous umbilical hernia repair and abdominoplasty. They specifically examined the laparoscopic, unilateral, and inferior midline techniques [26]. Due to the novelty of the topic at the time of the study, the studies reviewed were few and did not include other ventral hernia types (e. g. incisional and epigastric). In addition, other emerging techniques were not reviewed. The current review extends the previous review by Person et al. by the inclusion of more recent articles, and inclusion of reports of the pre-rectus, distal bilateral and proximal bilateral approaches and a focus on investigating the translatability of current evidence to clinical practice according to OCEBM [19, 26]. All studies reviewed were found with poor-to-moderate-quality evidence.

Apart from effectiveness in preserving umbilical blood supply, other aspects such as advantages and limitations of techniques should also be considered when suggesting a recommendation for clinical practice or further studies. The description of these aspects was more readily available in the laparoscopic and pre-rectus approaches for their relatively higher number of five studies each, followed by the unilateral approach with three studies, and finally the distal bilateral, proximal bilateral and inferior midline approaches with single studies each.

The laparoscopic approach presents several advantages, the most significant being the allowance of an intraperitoneal mesh repair which reduces hernia recurrence rates compared to suture repair [7, 16, 17, 20]. It also avoids infective complications associated with open surgery [7, 20]. However, the laparoscopic approach is believed to be unsuitable for persons with high BMI and is associated with extended intraoperative time compared to open surgery [17, 20]. To address these controversies, it was found that the laparoscopic approach was still feasible in those with high BMI. Le Gall et al., Lari et al., and van Schalkwyk et al. performed a laparoscopic approach on subjects with the highest reported BMI of 45.1 kg/m^2^, 34.1 kg/m^2^, and 30.1 kg/m^2^, respectively, all of which were above the obesity cutoff of BMI 30 kg/m^2^ [7, 17, 20, 27]. Additionally, the laparoscopic approach was found to be performed within a similar or even shorter intraoperative time compared to open surgery when executed by experienced surgeons [17, 20].

The pre-rectus approach could also potentially reap the benefits of a mesh, fixed with the onlay technique as described by Sakr et al. and Eltantawy et al. [8, 22]. A reduction in hernia recurrence rate was proven by a decrease of 57.5% to 28.9% in Sakr et al., when comparing abdominoplasty alone to combined herniorrhaphy and abdominoplasty [8]. This is in unison with Eltantawy et al. who found no hernia recurrence [22]. Likewise, the unilateral, distal bilateral, and proximal bilateral approaches were described to utilize a mesh in their respective planes they provide access [3, 15, 23–25]. The inferior midline approach, although reported by a single study to use sutures for hernia repair, could theoretically also incorporate mesh fixation [10]. These techniques are open approaches that appeared to be preferred in patients with high BMI above 30 kg/m^2^, as reported in several studies [3, 6, 8, 9, 22, 24].

On the surface, the laparoscopic technique compared to the others appeared most promising in preserving umbilical blood supply. However, particular techniques cannot be recommended nor proven ineffective due to a lack of evidence in all techniques, each to a varying extent. Firstly, each technique was reported by few studies with a maximum of five each in the laparoscopic and pre-rectus approaches. Secondly, none of the studies had an alternate technique as a comparison. Lastly, some (five of 16) studies did not observe umbilical complications as an outcome of interest. This is further compounded by a limitation of this study in which preserving the umbilical blood supply was the main concern. It was recognized that other outcomes, including hernia recurrence rates, intraoperative time, infection rates, cosmesis and patient satisfaction rates, should contribute to the utility of techniques in clinical practice. On top of that, each technique appeared to present its respective pros and cons, suggesting that recommending a technique should be tailored to the patient.

To better assess each technique’s effectiveness, comparative studies are required. A systematic review provides the best evidence; however, this is unfeasible in the setting of low numbers of controlled trial studies and inconsistent reporting of outcomes among existing studies. A randomized controlled trial could be designed to compare the techniques, for example, comparing the effectiveness of the laparoscopic versus pre-rectus approach, and to better report the outcomes, the incidence and severity of complications.

Introducing abdominoplasty to ventral hernia repair potentially provides advantages; however, it is imperative to underscore that the efficient reconstruction of the abdominal wall should be prioritized over preserving umbilical vascularization. In instances where sacrificing the umbilicus is unavoidable and necessary, for example, umbilical hernias encroaching on the umbilical stalk, the failure to preserve the umbilicus is not a catastrophic outcome [22]. Furthermore, umbilical necrosis often heals well and is an aesthetic rather than a medical complication, which may be followed by umbiliconeoplasty [22, 28].

## Conclusion

The identified techniques lack high-quality evidence. Nonetheless, they remain viable options for combined ventral hernia repair and abdominoplasty. Therefore, large-scale high-quality RCTs are required to compare the effectiveness of various approaches with additional outcomes of hernia recurrence rates, intraoperative time, and patient- and surgeon-reported satisfaction.
